# Comparison of Camouflage Agents on Quality‐of‐Life Improvement in Vitiligo Patients

**DOI:** 10.1111/jocd.70949

**Published:** 2026-06-16

**Authors:** Yu Yan, Shutian Wu, Shiyan Wang, Jianhua Huang, Shimin Zhang, Jingjuan Shi, Lei Shi, Hongwei Wang, Xiuli Wang

**Affiliations:** ^1^ Institute of Photomedicine, Shanghai Skin Disease Hospital Tongji University School of Medicine Shanghai China; ^2^ Department of Dermatology Huadong Hospital Affiliated to Fudan University Shanghai China

**Keywords:** camouflage therapy, minimum erythema dose (MED), Sun protection factor (SPF), UV protection, vitiligo

## Abstract

**Background:**

Vitiligo profoundly compromises patients' quality of life, yet camouflage therapy—an important adjunctive intervention—remains underutilized. Clinical observations reveal marked differences in the performance of various camouflage agents under Wood's lamp examination, suggesting potential disparities in their ultraviolet (UV) protective capabilities. However, evidence‐based guidance for patients and clinicians regarding product selection is currently lacking.

**Objective:**

This study aimed to quantify the photoprotective properties of two agents (A/B) and assess the dual benefits of concealment plus photoprotection.

**Methods:**

A two‐phase prospective cohort study was conducted. First, 31 healthy volunteers evaluated minimal erythema dose (MED), sun protection factor (SPF), minimal persistent pigment darkening dose (MPPD), and UVA protection factor (UVAPF) for Agents A/B. Second, 36 progressive non‐segmental facial vitiligo patients were randomized to use Agent A or B for one month. Primary outcome was Dermatology Life Quality Index (DLQI) change from baseline. Within‐group changes were analyzed using paired t‐tests; between‐group post‐treatment DLQI was compared using ANCOVA with baseline DLQI as a covariate.

**Results:**

Agent A demonstrated significant UV protective efficacy (SPF = 29.87 ± 2.31; UVAPF = 3.93 ± 0.95; PA+), whereas Agent B exhibited no measurable photoprotection. Baseline DLQI scores showed no significant difference between groups (16.18 ± 2.94 vs. 15.71 ± 4.00; *p* = 0.70). After one month, both groups showed significant within‐group DLQI improvements. After adjusting for baseline DLQI using ANCOVA, the Agent A group demonstrated a trend toward greater improvement compared to the Agent B group (adjusted mean difference: 1.78, 95% CI: −0.01 to 3.57, *p* = 0.051), with improvement rates of 46.0% and 29.7%, respectively.

**Conclusion:**

Camouflage agents enhance vitiligo patients' quality of life. Agents combining concealment and sun protection provide dual benefits—immediate masking and enhanced photoprotection—and are associated with a trend toward superior short‐term outcomes. Clinicians should prioritize sun‐protective camouflage products in comprehensive management plans.

## 1 Introduction

Vitiligo is a chronic autoimmune disease characterized by the selective loss of melanocytes, resulting in depigmented skin patches [1]. As an acquired depigmentation disorder, it affects approximately 0.5%–2% of the global population [2]. The condition presents with distinctive white macules that primarily alter physical appearance and negatively impact body image. Compared to healthy individuals or patients with other dermatological conditions such as alopecia areata, atopic dermatitis, or psoriasis, individuals with vitiligo exhibit significantly higher risks of anxiety, depression, sexual dysfunction, and suicidal ideation or behavior [3, 4, 5, 6]. These psychosocial burdens severely compromise the quality of life of affected patients. Research identifies darker skin types, rapid disease progression, and facial involvement as definitive predictors of heightened stigma [7]. The disease often manifests before age 20, exposing patients to substantial stigma and psychosocial stress during critical personal and professional developmental stages [8]. Psychological stress itself has been confirmed as one of the triggers or exacerbating factors for vitiligo, thereby creating a vicious cycle between disease burden and psychological distress [9]. Approximately 50% of vitiligo patients experience psychological stress preceding skin depigmentation or disease onset [9]. This stress disrupts the neuroendocrine‐immune system balance [10]. Consequently, promoting facial lesion repigmentation or concealment has become an indispensable component of vitiligo management strategies [11, 12].

Regrettably, no cure for vitiligo currently exists. Conventional treatments—including pharmacotherapy, phototherapy, and surgical interventions—show variable and often unsatisfactory efficacy [13]. Results typically require several months to become apparent [14, 15]. Even after successful repigmentation, recurrence rates reach 44% in the first year post‐treatment cessation [16]. Treatment options are further limited for special populations like children and pregnant women.

While camouflage therapy may not directly treat the disease, it holds significant value in alleviating psychosocial burdens [17, 18, 19]. Over half of vitiligo patients routinely use cosmetics, concealers, or clothing to hide lesions [5]. However, camouflage therapy remains overlooked and underestimated in clinical practice [20]. Only 21.6% of dermatologists discuss camouflage options with patients, and over half of users report difficulty finding suitable agents [21].

**FIGURE 1:**
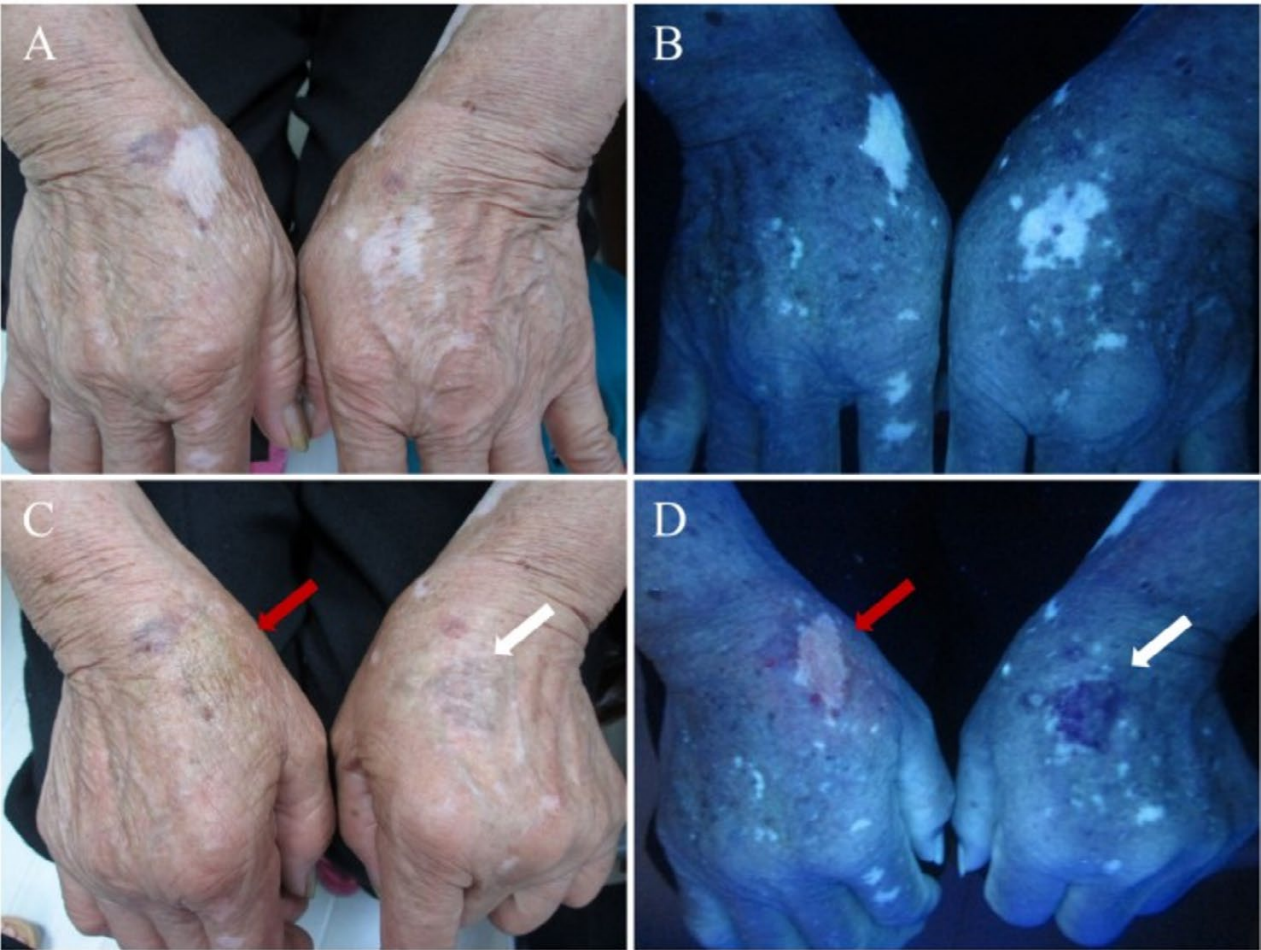
Camouflage Efficacy of Two Camouflage Agents. A: Vitiligo lesions under natural light. B: Vitiligo lesions exhibiting bright porcelain‐white fluorescence under Wood's lamp. C: Post‐application under natural light: Both camouflage agent A (white arrows) and agent B (red arrows) effectively conceal lesions with no visible white patches. D: Post‐application under Wood's lamp: Agent A (white arrows) demonstrates superior camouflage efficacy compared to agent B (red arrows).

In clinical practice, we observed that while both camouflage agents A and B effectively concealed lesions under natural light, their performance differed markedly under Wood's lamp (a filtered UV lamp) (Figure 1). This suggests superior UV protection in agents with better concealment. Excessive UV exposure induces Koebner phenomenon in progressive vitiligo, damaging melanocytes [22, 23] and exacerbating lesions. With 82% of patients exhibiting heightened sun sensitivity in depigmented areas (prone to sunburn) [24], UV protection is critical. Yet, studies exploring the added benefits of UV‐protective camouflage agents remain scarce.

To address this gap, we conducted a prospective cohort trial. First, UV protection performance was assessed in vitro using 31 healthy volunteers. Only agent A demonstrated significant UV protection (SPF = 29.87 ± 2.31; UVAPF = 3.93 ± 0.95). A subsequent trial enrolled 36 patients with facial vitiligo. Both groups showed significant QoL improvements (DLQI scores) after 1 month (*p* < 0.05), and agent A showed a trend toward greater improvement after adjusting for baseline DLQI (adjusted *p* = 0.051).

In conclusion, camouflage agents serve as effective adjuvants for improving QoL in vitiligo patients, with UV‐protective agents offering enhanced benefits. Clinicians should prioritize UV‐protective camouflage agents to simultaneously conceal lesions, provide photoprotection, and optimize QoL and disease management outcomes.

## 2 Methods

### 2.1 Study Participants

This study was approved by the Medical Ethics Committee of Shanghai Skin Disease Hospital. 31 healthy volunteers were recruited to evaluate the UV protection efficacy of camouflage agents A and B. Additionally, 36 patients with progressive non‐segmental facial vitiligo (confirmed by attending dermatologists using the 2021 Chinese consensus on the diagnosis and treatment of vitiligo [25]) were enrolled. Inclusion criteria: patients without prior camouflage agent use. Exclusion criteria: incomplete medical records; suspected non‐vitiligo pigmentary disorders; history of photosensitivity disorders; UV exposure contraindications; skin cancer, or cardiac/hepatic/renal dysfunction; pregnancy or lactation. Healthy volunteer exclusion criteria: (1) history of skin cancer, photosensitivity, or photosensitive dermatoses; (2) use of photosensitizing medications within 7 days; (3) erythema, dyspigmentation, scars, or tattoos at test sites; (4) chronic dermatoses (e.g., psoriasis, hyperpigmentation, lichen planus, scars, eczema).

### 2.2 Randomization, Intervention, and Sample Size

Baseline Dermatology Life Quality Index (DLQI) scores were obtained for all participants. Eligible patients were randomly assigned (1:1) to receive either Agent A or Agent B using a computer‐generated random number table. Allocation concealment was achieved using sequentially numbered, opaque, sealed envelopes. Due to the visible differences in physical properties between the two camouflage agents, blinding of patients and the outcome assessor was not feasible. Patients applied the assigned agent once daily for 1 month. Follow‐up DLQI scores were collected via telephone interviews at 1 month post‐application [26].

Sample size justification: This was an exploratory pilot study. No prior literature provided reference values for expected DLQI differences or standard deviations specifically for camouflage agents with UV protection. Therefore, a formal sample size calculation was not performed; the sample size (*n* = 36) was determined based on feasibility and clinical availability. We acknowledge this as a limitation and recommend future confirmatory studies with larger sample sizes.

### 2.3 UV Protection Efficacy

#### 2.3.1 Equipment

A solar UV simulator (280–320 nm filtered output) was used.

#### 2.3.2 Methodology

Three adjacent 10 cm × 2 cm test sites were marked on the upper back of 17 healthy volunteers. Sites were assigned as: experimental (agent A, 2.0 mg/cm^2^), control (agent B, 2.0 mg/cm^2^), and untreated control. Eight 1 cm‐diameter UV spots (6‐min exposure) were applied, with energy density decreasing by 20% between adjacent spots. Erythema was documented 24 h post‐exposure via digital photography under natural light.

### 2.4 Determination of MED, SPF, MPPD, UVAPF, and PA

#### 2.4.1 Equipment

Solar UV simulator (as per 2.3.1).

#### 2.4.2 Methodology

MED/SPF Assessment (14 volunteers): Test sites (10 cm × 2 cm) were marked on the right and left infrascapular regions. The right site received agent A (2.0 mg/cm^2^); the left served as untreated control. Eight UV spots (20% energy reduction between spots) were applied. MED was determined 24 ± 2 h post‐exposure by two blinded dermatologists. SPF = protected MED/unprotected MED.

MPPD/UVAPF/PA Assessment: Test sites (10 cm × 2 cm) were marked on the left and right abdominal regions. The left site received agent A (2.0 mg/cm^2^); the right served as untreated control. MPPD (minimal persistent pigment darkening) was assessed 2–4 h post‐exposure. UVAPF = protected MPPD/unprotected MPPD. PA grading was derived from UVAPF values (Table S1).

### 2.5 Statistical Analysis

Data were analyzed using IBM SPSS Statistics 23.0 (SPSS Inc., USA). Continuous variables were expressed as mean ± standard deviation (SD) or median with interquartile range (IQR) as appropriate. Normality of continuous variables (age, disease duration, and DLQI scores) was assessed using the Shapiro–Wilk test.

For baseline comparisons between Agent A and Agent B groups, normally distributed continuous variables were analyzed using independent samples *t*‐test; non‐normally distributed variables were analyzed using the Mann–Whitney U test. Categorical variables (sex, Fitzpatrick skin type) were compared using the chi‐square test or Fisher's exact test, as appropriate.

The primary outcome was change in Dermatology Life Quality Index (DLQI) from baseline to 1 month. Within‐group pre‐post differences were evaluated using paired *t*‐tests. To compare post‐treatment DLQI between groups while adjusting for baseline DLQI, analysis of covariance (ANCOVA) was performed with baseline DLQI as a covariate. Estimated marginal means with 95% confidence intervals (CIs) and adjusted mean differences are reported.

This was an exploratory pilot study without prior effect size estimates; therefore, a formal sample size calculation was not performed. Statistical significance was defined as two‐sided *p* < 0.05 (*), *p* < 0.01 (**), *p* < 0.001 (***), and *p* < 0.0001 (****).

## 3 Results

### 3.1 UV Protection Efficacy of Two Camouflage Agents

Table S2 presents demographic data of 17 healthy volunteers (mean age 33.88 ± 5.30 years, range 28–44; 58.82% male). Fitzpatrick skin types: III (29.41%), IV (70.59%).

**FIGURE 2:**
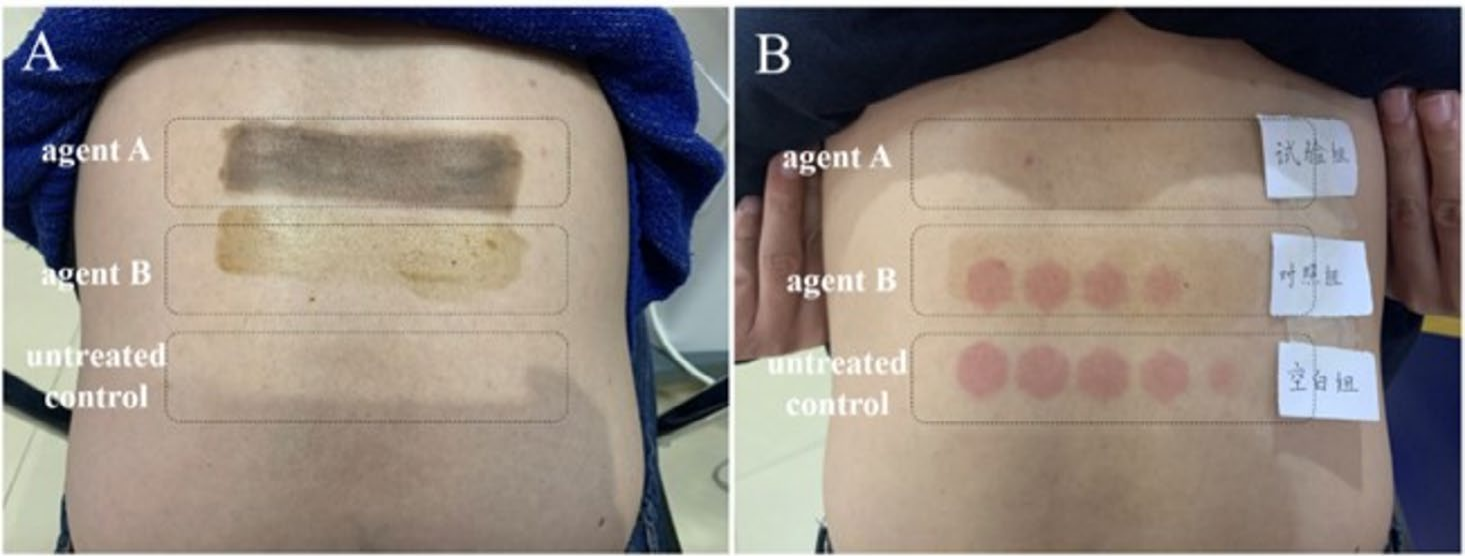
UV Protection Efficacy of Camouflage Agents. A: Pre‐irradiation dorsal skin sites treated with agent A, agent B, and untreated control. B: Post‐irradiation erythema responses in experimental, control, and untreated sites.

In Figure 2, the agent A‐treated experimental sites showed no erythema across all UV spots, while the agent B‐treated control sites exhibited significant erythema correlated with energy density. Agent B's MED (675.44 ± 105.93 mJ/cm^2^) showed no significant difference from untreated controls (619.16 ± 136.75 mJ/cm^2^; *p* = 0.083).

### 3.2 MED, SPF, MPPD, UVAPF, and PA of Agent A

Table S3 summarizes 14 volunteers (mean age 38.79 ± 9.84 years, range 24–59; 64.29% male). Fitzpatrick skin types: III (42.86%), IV (57.14%).

**FIGURE 3:**
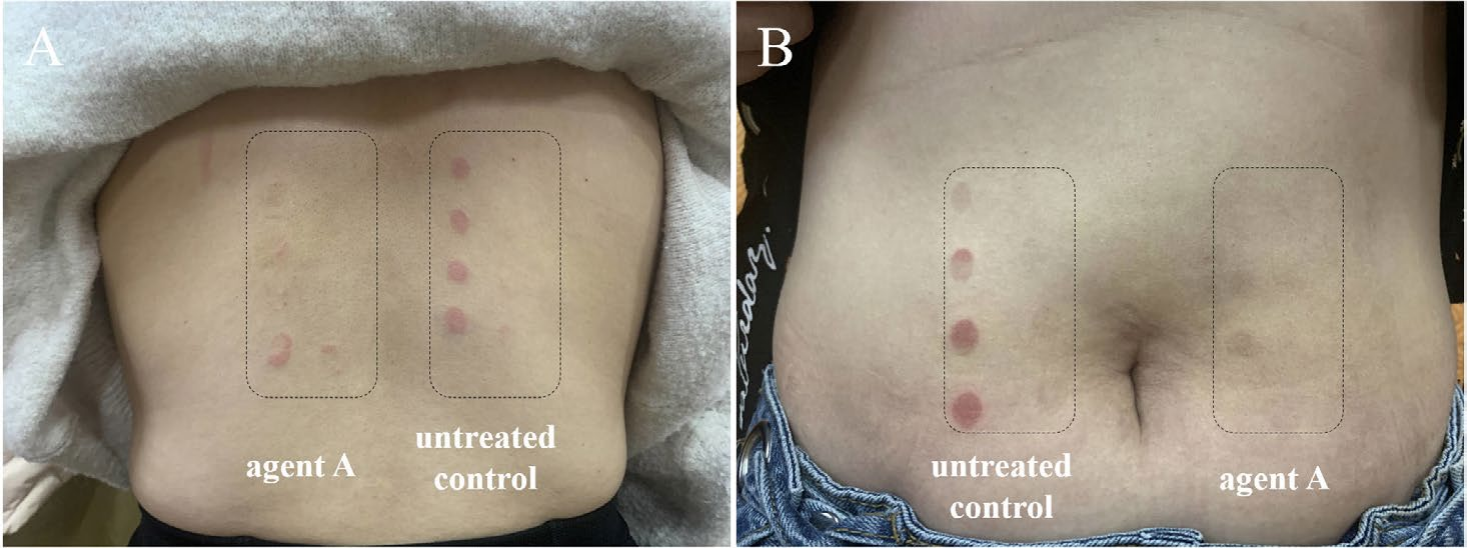
MED and MPPD Determination for Agent A. A: Erythema responses 24 ± 2 h post‐irradiation in treated vs. untreated sites. B: Pigment darkening responses 2–4 h post‐irradiation.

Camouflage agent A demonstrated significantly superior UV protection efficacy compared to untreated controls, as evidenced by key photoprotection metrics (Figure 3). The MED for agent A was 5800.93 ± 1626.31 mJ/cm^2^, which was statistically significantly higher than the untreated control MED of 193.86 ± 49.85 mJ/cm^2^ (t = 13.29, *p* < 0.0001). Similarly, the MPPD for agent A was 134.42 ± 40.55 mJ/cm^2^, showing a statistically significant difference compared to the untreated control MPPD of 34.17 ± 11.77 mJ/cm^2^ (t = 10.06, *p* < 0.0001). Additional photoprotection parameters included a SPF of 29.87 ± 2.31 and a UVAPF of 3.93 ± 0.95, corresponding to a PA grade of +, indicating minimal UVA protection. These results collectively confirm the enhanced photoprotective efficacy of agent A in mitigating UV‐induced skin damage.

### 3.3 QoL Outcomes

Of 36 enrolled progressive non‐segmental facial vitiligo patients, 2 were lost to follow‐up in the agent B group (Figure S1). Baseline characteristics are shown in Table 1. The Shapiro–Wilk test confirmed normal distribution for age and baseline DLQI (Agent A: *p* = 0.46; Agent B: *p* = 0.09), while disease duration was not normally distributed (*p* = 0.002).

**TABLE 1:** Baseline patient demographics (*n* = 34).

Characteristic	All patients (*n* = 34)	Agent A (*n* = 17)	Agent B (*n* = 17)	*p*
Age (years)^a^	43.4 ± 14.4	43.4 ± 15.3	43.5 ± 15.3	0.97
Gender, *n* (%)^c^	Male: 19 (55.9)	Male: 10 (58.8)	Male: 9 (52.9)	0.73
	Female: 15 (44.1)	Female: 7 (41.2)	Female: 8 (47.1)	
Disease duration (months)^b^	12.5 [6–18]	11 [5–26]	15 [12–17]	0.65
Fitzpatrick skin type, *n* (%)^c^	Type III: 11 (32.4) Type IV: 23 (67.6)	Type III: 5 (29.4) Type IV: 12 (70.6)	Type III: 6 (35.3) Type IV: 11 (64.7)	0.71
Baseline DLQI^a^	15.9 ± 3.5	16.2 ± 2.9	15.7 ± 4.0	0.70

^a^ Independent *t*‐test.

^b^ Mann–Whitney *U* test (non‐normal distribution).

^c^ Mann–Whitney *U* test.

Baseline DLQI scores showed no significant difference between groups (Agent A: 16.2 ± 2.9, Agent B: 15.7 ± 4.0; independent *t*‐test, *p* = 0.70). Both groups showed significant within‐group improvement from baseline (Agent A: 8.8 ± 1.9, paired *t*‐test, *p* < 0.0001; Agent B: 10.9 ± 3.3, paired *t*‐test, *p* = 0.001).

To compare the magnitude of improvement between groups while adjusting for baseline DLQI, ANCOVA was performed with baseline DLQI as a covariate. The estimated marginal mean post‐treatment DLQI was 8.99 (95% CI: 8.33–9.65) in the Agent A group and 10.77 (95% CI: 10.11–11.43) in the Agent B group, with an adjusted mean difference of 1.78 (95% CI: −0.01 to 3.57, *p* = 0.051). Thus, the Agent A group showed a trend toward greater improvement that did not reach conventional statistical significance. The improvement rates were 46.0% and 29.7%, respectively.

## 4 Discussion

Vitiligo, recognized as a psychosomatic dermatosis, significantly impacts mental health and QoL [5, 27]. Research indicates that approximately 75% of vitiligo patients experience psychological comorbidities [28], with depression being the most prevalent (62.29%) [29]. Anxiety risk in this population is 6.14 times higher than in healthy controls [30]. In the present study, 94% of patients exhibited DLQI scores > 10, confirming severe QoL impairment, often manifesting as moderate‐to‐severe distress over appearance deficits, reduced self‐esteem, social isolation, and occupational limitations [31, 32]. While existing treatments may partially control or reduce lesions and promote repigmentation, they frequently suffer from slow onset, prolonged duration, and high recurrence rates, particularly in progressive disease, failing to meet patient expectations in the short term [33, 34]. Physician‐reported treatment satisfaction rate is 56%, and 25% of vitiligo patients report frustration with the treatment regimen [35]. During extended treatment phases, camouflage therapy emerges as a necessary and effective adjuvant, utilizing waterproof, opaque products that employ light absorption/reflection principles to match surrounding skin tone, thereby improving appearance and psychosocial integration [36]. Despite mentions in international guidelines with low recommendation strengths (C/D grade) [37, 38, 39], camouflage remains underutilized in clinical practice.

Camouflage therapy employs waterproof, opaque products that utilize light absorption and reflection principles to achieve natural skin tone matching, thereby concealing disfiguring lesions [40]. This approach has been extensively applied in burn rehabilitation and dermatoses such as alopecia areata, acne, and scars [18, 41]. A wide variety of camouflage agents exist, primarily consisting of cosmetic concealers like foundation creams, lotions, and covering solutions [42], which exhibit significant variability in both camouflage efficacy and UV protection.

Prior to this study, clinical observations revealed that while both agents A and B effectively concealed vitiligo lesions under natural light, Wood's lamp examination demonstrated distinct differences: lesions covered with agent A showed no porcelain‐white fluorescence, whereas agent B‐treated areas retained visible depigmentation, suggesting UV‐protective properties for agent A (Figure 1). Excessive UV exposure induces melanocyte toxicity and programmed necrosis, exacerbating vitiligo progression [43, 44, 45]. A prospective cohort study of 51 337 women confirmed that severe sunburn increased vitiligo risk 2.17‐fold in fair‐skinned individuals [46], with sunburn being the primary physical manifestation of the disease [24]. Although brief outdoor UV exposure (10–15 min) may sufficiently enhance response to topical treatments [47], vitiligo patients should be encouraged to use sun protection measures [4, 48]. This helps prevent sunburn and reduces the contrast between affected and unaffected skin areas.

Our findings revealed significant disparities in UV protection between the two agents. While agent B showed no significant MED difference from untreated controls (*p* = 0.083), agent A demonstrated markedly superior photoprotection: MED (5800.93 ± 1626.31 mJ/cm^2^ vs. 193.86 ± 49.85 mJ/cm^2^) and MPPD (134.42 ± 40.55 mJ/cm^2^ vs. 34.17 ± 11.77 mJ/cm^2^) values were significantly higher (*p* < 0.0001 for both), with SPF 29.87 ± 2.31, UVAPF 3.93 ± 0.95, and PA grade +. Both agents improved DLQI scores after 1 month (agent A: 8.8 ± 1.9, *p* < 0.0001; agent B: 10.9 ± 3.3, *p* = 0.001). After adjusting for baseline DLQI using ANCOVA, agent A showed a trend toward greater improvement (adjusted mean difference: 1.78, 95% CI: −0.01 to 3.57, *p* = 0.051).

During prolonged treatment and repigmentation phases, camouflage therapy offers rapid, convenient lesion concealment, reducing anxiety and facilitating psychosocial reintegration. UV‐protective agents may provide dual benefits: immediate aesthetic improvement and potential disease stabilization by mitigating sun‐induced damage, thereby optimizing lesion protection. However, confirmatory studies are required to establish these benefits definitively.

### 4.1 Interpretation of Quality‐Of‐Life Findings

While UV protection may contribute to the observed trend toward greater improvement in the Agent A group, other factors cannot be excluded. Agent A might have provided superior natural‐light concealment, better skin texture match, or higher patient satisfaction with its cosmetic qualities, independent of its UV‐protective properties. Our study was not designed to establish causality, and the results should be interpreted as hypothesis‐generating.

### 4.2 Limitations

This study has several limitations. First, it was an exploratory pilot study without a formal sample size calculation; the limited sample (*n* = 34 completers) reduces statistical power. Second, the between‐group difference after ANCOVA did not reach statistical significance (*p* = 0.051), which may reflect insufficient power rather than absence of effect. Third, the one‐month follow‐up is too short to assess long‐term safety, tolerability, or willingness to continue using the product. Fourth, the UV protection evaluation was conducted on a small number of healthy volunteers (*n* = 14–17) with Fitzpatrick skin types III–IV only, limiting generalizability to lighter skin types (I–II) or other populations. Fifth, photoprotection durability under real‐world conditions (e.g., after sweating, rubbing, or long‐term wear) was not assessed. Sixth, due to the visible differences between the two agents, blinding was not feasible, introducing potential performance bias. Finally, the lack of mediation analysis prevents disentangling the relative contributions of UV protection versus cosmetic acceptability to the DLQI improvement. Larger, blinded, randomized controlled trials with longer follow‐up and diverse skin types are needed to confirm the dual‐benefit concept.

## Author Contributions

Y.Y.: conceptualization, methodology, formal analysis, writing – original draft. S.W.: methodology, investigation, data curation, writing – original draft. S.W.: investigation, resources, validation. J.H.: resources, supervision, writing – review and editing. S.Z.: methodology, software, formal analysis. J.S.: validation, visualization, project administration. L.S.: resources, data curation, writing – review and editing. H.W.: conceptualization, supervision, writing – review and editing. X.W.: conceptualization, supervision, writing – review and editing.

## Funding

The authors have nothing to report.

## Ethics Statement

Reviewed and approved by the Ethics Committee of Shanghai Skin Disease Hospital.

## Consent

The authors confirm that all patients provided written informed consent for the publication of identifiable material, with the clear understanding that this information may be publicly available.

## Conflicts of Interest

The authors declare no conflicts of interest.

## Supporting information


**Table S1:** UVAPF and PA Correspondence Table.
**Table S2:** Demographics of Healthy Volunteers in UV Protection Study.
**Table S3:** Demographics for Agent A Efficacy Testing.
**Figure S1:** Flow chart of the study.

## Data Availability

The data that support the findings of this study are available on request from the corresponding author. The data are not publicly available due to privacy or ethical restrictions.
