# Supplementary material for: Comparison of Camouflage Agents on Quality‐of‐Life Improvement in Vitiligo Patients
Source: J Cosmet Dermatol. 2026 Jun 16;25(6):e70949. doi: 10.1111/jocd.70949 (PMC13270214; doi:10.1111/jocd.70949)
Supplement: Supplementary file 1 — Table S1: UVAPF and PA Correspondence Table. Table S2: Demographics of Healthy Volunteers in UV Protection Study. Table S3: Demographics for Agent A Efficacy Testing. Figure S1: Flow chart of the study. [file JOCD-25-e70949-s001.docx]

Supplementary Table 1: UVAPF and PA Correspondence Table

| UVAPF | PA |
| --- | --- |
| 2 ～ 4 | + |
| 4 ～ 8 | ++ |
| 8 ～ 16 | +++ |
| ≥16 | ++++ |

Supplementary Table 2. Demographics of Healthy Volunteers in UV Protection Study

| Characteristic | Value |
| --- | --- |
| Age (years) | Mean ± SD: 34.1±5.3; Range: 28–44 |
| Gender, n (%) | Male: 11 (64.7); Female: 6 (35.3) |
| Fitzpatrick Skin Type, n (%) | Type III: 6 (35.3); Type IV: 11 (64.7) |

Supplementary Table 3. Demographics for Agent A Efficacy Testing

| Characteristic | Value |
| --- | --- |
| Age (years) | Mean ± SD: 38.6±9.5; Range: 24–59 |
| Gender, n (%) | Male: 9 (64.3); Female: 5 (35.7) |
| Fitzpatrick Skin Type, n (%) | Type III: 6 (42.9); Type IV: 8 (57.1) |


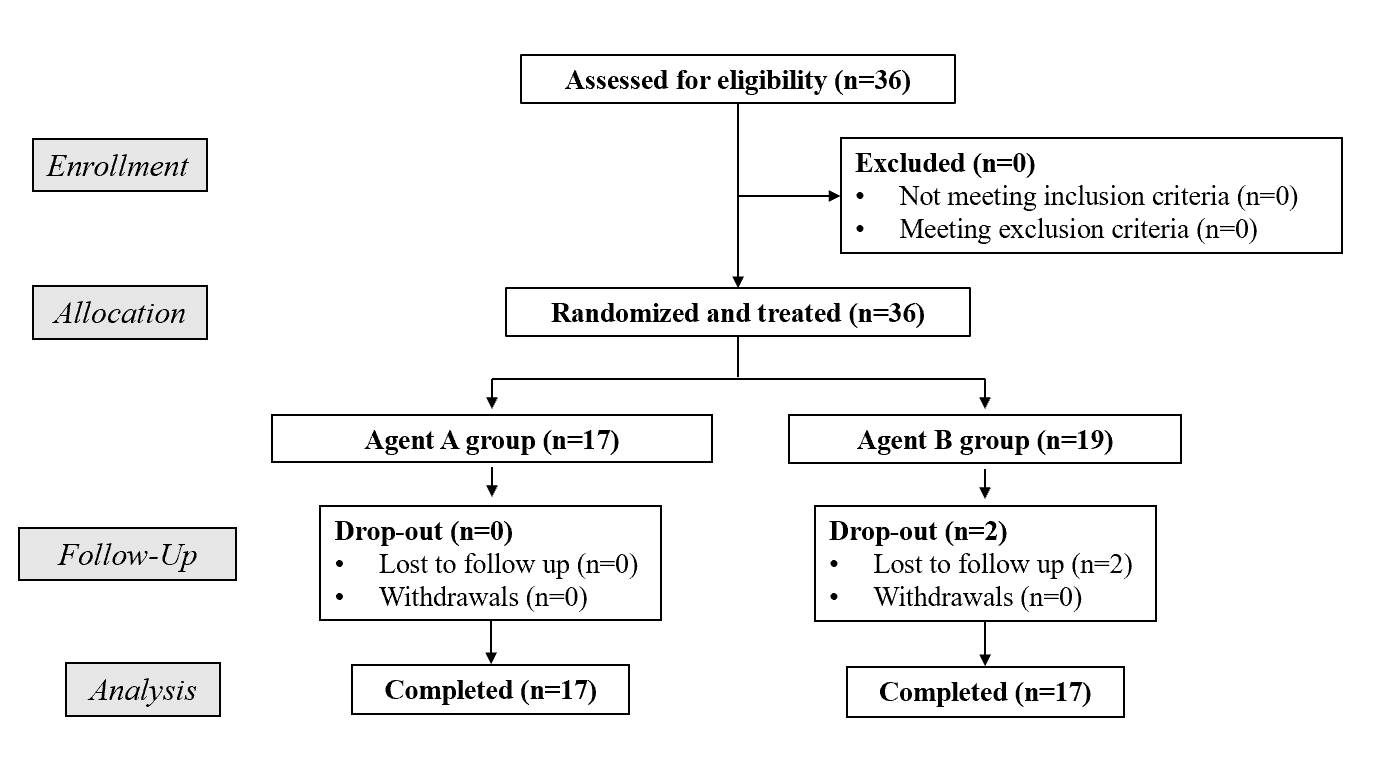


Supplementary Figure 1. Flow chart of the study.
